# Forecasting the need for medical specialists in Spain: application of a system dynamics model

**DOI:** 10.1186/1478-4491-8-24

**Published:** 2010-10-29

**Authors:** Patricia Barber, Beatriz González López-Valcárcel

**Affiliations:** 1 University of Las Palmas de Gran Canaria, Campus Universitario de Tafira, 35017 Las Palmas de G.C., Canary Islands, Spain

## Abstract

**Background:**

Spain has gone from a surplus to a shortage of medical doctors in very few years. Medium and long-term planning for health professionals has become a high priority for health authorities.

**Methods:**

We created a supply and demand/need simulation model for 43 medical specialties using system dynamics. The model includes demographic, education and labour market variables. Several scenarios were defined. Variables controllable by health planners can be set as parameters to simulate different scenarios. The model calculates the supply and the deficit or surplus. Experts set the ratio of specialists needed per 1000 inhabitants with a Delphi method.

**Results:**

In the scenario of the baseline model with moderate population growth, the deficit of medical specialists will grow from 2% at present (2800 specialists) to 14.3% in 2025 (almost 21 000). The specialties with the greatest medium-term shortages are Anesthesiology, Orthopedic and Traumatic Surgery, Pediatric Surgery, Plastic Aesthetic and Reparatory Surgery, Family and Community Medicine, Pediatrics, Radiology, and Urology.

**Conclusions:**

The model suggests the need to increase the number of students admitted to medical school. Training itineraries should be redesigned to facilitate mobility among specialties. In the meantime, the need to make more flexible the supply in the short term is being filled by the immigration of physicians from new members of the European Union and from Latin America.

## Background

The provision of human resources in the health field is a logistical task of great complexity. The need for long-term planning in a context of uncertainty and on a national scale, the interconnections between training, formal position and actual duties, and tensions over jurisdiction between national and regional authorities aggravate the problem. The labour market for health professionals must be extremely adaptable in order to absorb swiftly changes required by new technologies, scientific advances, societal demands, and new models of organization. The job profiles of health specialists, however, have not been adapting to this rapid and exigent pace of change.

A shortage of health professionals, whether because of poor planning or corporative barriers to entry in the profession, appears to be a problem in many developed countries. Planning for health human resources has become a high priority for OECD countries[1]; it was the focus of the World Health Organization (WHO) annual World Health Report for 2006[2]; and at present it is high on the international agenda, with the EU "Green Paper on the European Workforce of Health" [3] and the EU Prometheus research project [4]. In Spain, perceived specialist shortages led the Health Ministry to ask the authors of this paper for a detailed study of the imbalances in the medical labour market in 2005 [5]. The study was updated in 2009 [6]. This article is based on the reports we submitted.

The task of planning human health resources consists in identifying and locating the right number of doctors with the appropriate specialties for the right place at the right time. The 'invisible hand' of the market and the 'stern hand' of government regulation are the tools that governments use, in differing proportions, to achieve this goal. Since there are groups lobbying on both sides, and the matter must be addressed with scientific neutrality, avoiding short-term solutions that are abandoned when the crisis has passed.

A dynamic system is almost always in disequilibrium. The important thing is to know it is on the right track. The challenge of dynamically adjusting the supply and demand of doctors involves making the right decisions at the right time about the number of slots for training, about retention and retirement of doctors in practice, and in regard to medical immigration; ensuring a reasonable composition of specialties and a balanced geographical distribution; and setting the right working conditions and compensation schedules. The planning methods we used are based on 'need,' 'demand' (use), or 'benchmarking' [7].

This planning is additionally complicated because the skill-mix that doctors need changes as their professional roles change and medical organizations change [8,9]. Globalization, which accelerates and multiplies international mobility and delocalizes some medical services, also makes planning more difficult [10], as it opens nations to external markets. International mobility has a substantial and growing impact on the market for doctors, one that is influenced by both push and pull forces and can at the same time be a problem and a solution [11]. It is useless to limit planning to a national territory, because the trend toward international mobility is irreversible.

There is no perfect method for planning for medical doctors [12]. None of the various methods has been applied in a pure form, although Australia [13-15], Canada [16-19], Germany, France, Netherlands and the United Kingdom have a long history and valuable experience with 'need-based' planning. The United States is a good example of medical assignment based on demand and the market, but in practice this approach is mixed with what is known as the 'professional' model, by which doctors control the entry into the profession and evaluate practice.

In Spain, too, medical professional associations have a say in decisions about the number of specialists to be trained, and in this sense it shares with the United States aspects of the 'professional' model. Health organization in Spain is based on the National Health System, which is fully funded by taxes, with universal coverage and without co-payment (apart from for certain few exceptions such as medicines). From the year 2002, the organization and administration of health is completely decentralized in Spain's seventeen Autonomous Communities. Decentralization of health services began in 1981 with Catalonia and took twenty years to complete; in 2001 and 2002 the state devolved health authority to the last ten communities.

Spain has a population of 46 million people. From 2000 to 2008, due to liberal immigration policies, it had the highest population growth rate of the European Union, with an average annual increase of 1.6% and a total increase of 15%, leading to a great increase in the need for health services, particularly those that are income-sensitive. In this expansive phase, Spain imported physicians from Eastern Europe and above all Latin America. The immigration of doctors, for Spain a relatively recent phenomenon, has reduced the tension between supply and demand, but has also led to professional, social and political controversy.

This study will present a method based on system dynamics for planning for human professional resources in the health sector, and will show how it was applied to physicians in Spain. Our model simulates the evolution of supply and demand of physicians in a predictive timeline up to 2025 for each of the 43 medical specialties. It permits the modification of inputs under government discretion (enrollment limits, specialist training positions, retirement age, etc.), and indicates the various possible vectors of the future evolution of supply and demand of medical specialists under different scenarios of government regulation, technology and demography.

### Planning for reducing imbalances in the supply of health professionals in Spain

In Spain there is an intense debate within the medical profession and in society in general about whether to adjust the enrolment of medical students [20], in a context of a disequilibrium [21] between the professions--a low ratio of nurses to doctors--a disequilibrium among specialists, and a moderately uneven geographic distribution of physicians. Some specialties have a top-heavy age distribution, which will lead to a problem of generational replacement in ten or fifteen years that will be difficult to resolve with the current rates of specialist training residencies [22].

On the supply side there are worries about an increased deficit in physicians. One reason is the feminization of the profession (two of every three new doctors are women), which entails a reduction in the total effective workweek, which is also being cut back for sociological and legal reasons. An increased appreciation for leisure time is a pattern common to physicians and other professionals, in Spain and elsewhere. Professionals demand new and better working conditions: flexible schedules, the possibility of part-time work in certain periods and of vacation time in segments. The number of hours that doctors work per week varies significantly between countries, but there is a general trend towards reduction [1,23]. Although the aging of the physician population does not seem to be a problem overall, the traditional specialties are quite over-age. In recent years the supply of doctors in the public health system has been sapped by a dynamic private sector, which has absorbed much of medical employment. Spain has experienced an unprecedented increase in private medical plans, financed by agreements with the state health system, private insurance policies, direct payments or by way of insurance of foreign patients, and direct out-of-pocket payments by patients who are Spanish residents. Furthermore, beginning in 2000 many Spanish doctors left to work in other EEU countries, particularly the United Kingdom but also France and Portugal, where the salaries and the working conditions were better. The chain of international mobility was completed by the arrival of Latin American physicians, attracted by better working conditions and a common language.

On the demand side, the underlying causes that have affected need for certain kinds of specialists include demographic growth and the aging of the population, which will particularly increase the need for geriatricians, urologists, and family practitioners. In spite of the depopulation of rural areas, a minimum number of doctors must be maintained there for reasons of equity. Furthermore, medical technology increases the need for specialists because of new procedures (such as catheterization in cardiology and new kinds of treatment in oncology) or to treat new illnesses. Although some new technologies replace human labour by mechanization (as in clinical analysis or computerization of information), in general, advances in health technology have been labour intensive, and many new techniques do not replace work but rather create new things for doctors to do. Some technologies permit delocalization, which is already beginning in medicine. For example, x-ray results can be transmitted by the internet to highly specialized centres, geographically concentrated [24], for evaluation. Changes in patterns of morbidity require changes in specialists; for example, diseases new to Spain have entered with the influx of immigrants. And finally, since the decentralization of the Health Service, Autonomous Communities have invested in new hospitals to improve access for their populations, and these in turn must be staffed with specialists.

Ways must be found to pay differential salaries in the public system, where the rigid labour legislation has meant that rural zones and small cities bear the brunt of the deficit in doctors. With its uniform salaries the public sector is less free than the private sector to compensate for the unevenness of supply and demand by economic incentives. International mobility has provided flexibility for the system over the short term. In an open system, international migratory flows attract doctors to some countries and repel them from others. Spain has joined this process of medical internationalization in the last decade.

## Materials and methods

### Data

One of the main problems the Spanish government faced in dealing with the present imbalances in the medical labour market is the absence of a register of medical professionals and their characteristics: specialty, age, gender, etc. A number of official and unofficial sources provide information, but not detailed enough for planning. The official survey of hospitals gives the number of physicians, but broken down only into four groups of specialists, and with no information on age. Professional organizations publish information on their members, but not by specialty, and in various Autonomous Communities membership in these organizations is not mandatory, so the number of doctors is underreported. Finally, the medical associations of different regions count differently those professionals who are retired from active practice.

Specifically for this study, and in a specially-designed format, all the regional health departments provided the Health Ministry with homogenous and complete data on its employed physicians by specialty, gender, and age group, with a reference date of July 2007. In addition, the Health Ministry provided detailed information on approximately 20 000 doctors in specialty training (MIR), on the choices of MIR positions from 1990 to 2008, and on the foreign doctors certified for practice in Spain, whether or not in the regional health systems.

In spite of the wealth of information for the public health system, the total number of doctors, including those in private practice, potentially or in fact active by specialty, gender and age group and the corresponding age pyramids, has had to be estimated ('reconstructed') from the fragmentary reports of the professional associations, official statistics (ESSCRI), the Survey of Active Population, reports of the Autonomous Communities' health services and planning commissions, and reports for some of the specialties [25]. Then the data was adjusted to calculate full-time professionals using estimated conversion rates for Spain [26].

The population projections and general mortality rates used were from the National Institute of Statistics.

At the request of the Health Ministry, some Autonomous Community health services provided data on the specialist positions that could not be filled for lack of applicants. In order to evaluate the present deficit of physicians by specialty, we also analyzed the job openings listed on the internet of all the medical societies.

To determine the standards for the present and future need for specialists in Spain (the ratio of full-time equivalent doctors per 100 000 population), the Ministry of Health made a Delphi-type two-phase consultation of experts named by the Ministry and Autonomous Community health authorities.

### The simulation model

Most of the published papers on physician workforce have studied particular specialties and populations in specific areas [27-30]. There are several methods for planning and projecting health human resources [31], including regression-based models [32], simulation models [33-35] and Markov chains [36]. We have designed and implemented a dynamic simulation model based on the system dynamics method developed by Forrester in 1958 [37,38] and since then frequently used in a wide variety of contexts [38], including human resources planning [39-43]. In Spain, system dynamics has been applied for designing long-term policies related to waiting lists in public hospitals [44] and to model medical practice variations among hospitals, focusing on organizational learning [45]. We used specialized software, Powersim Studio 2005, for the implementation of these models. The model is a user-friendly tool for physician workforce planning.

The structure of a system, the relationships that exist between its variables, works over time to produce dynamic behaviour patterns of the system's variables. The objective of System Dynamics models is to understand how the structure of a system determines its behaviour. This understanding normally produces a framework for determining what actions can improve the system or fix its problems. In a system dynamics model, the simulations are essentially time-step simulations. The model takes a number of simulation steps along the time axis.

System Dynamics makes extensive use of diagrams, especially of two types: causal loop, and stock and flow.

### Causal loop

A causal-loop diagram identifies the structures and interactions of feedback loops, and consists of variables for cause and effect, and causal links. A causal link connects a cause variable with an effect variable by a link with a positive or negative charge. A positive link from variable X to variable Y means either that X adds to Y or that a change in X results in a change to Y in the same direction. A negative link from X to Y means either that X subtracts from Y or that a change in X results in a change in Y in the opposite direction [46]. Causal loops can be reinforcing (if, after going around the loop, it ends up with the same result as the initial balancing) or balancing (if the result contradicts the initial assumption). Loops with positive-feedback are associated with explosive growth, while loops with negative feedback tend to equilibrium. Loops can be nested, and they can also be affected by delayed relationships among variables. Those characteristics ultimately determine the evolutionary path-logistic, oscillatory or otherwise-of the loops [46-49].

### Stock and flow

Stock and flow diagrams are building blocks for models for quantitative analysis of system dynamics behaviour, and they have two kinds of variables.

Stock or levels variables describe the states of the system, such as the number of medical specialists, while flow variables depict the rates of change of levels, such as the number of training positions that are available. Stocks are accumulations of flows, and are calculated mathematically as the integration of net inflows [50], i.e.,

$$Stock(t)=\int_{0}^{t}\left[Inflow-Outflow]ds+Stock(t_{0}\right)$$

with Inflow(s) and Outflows(s) denoting the values of the inflow and outflow at any time s between the initial time and the present time t. Conversely, the net flow determines the rate of change of any stock, i.e. its time derivative, by the differential equation [50]:

$$\frac{d(Stock)}{dt}=Inflow(t)-Outflow(t)$$

**Figure 1:**
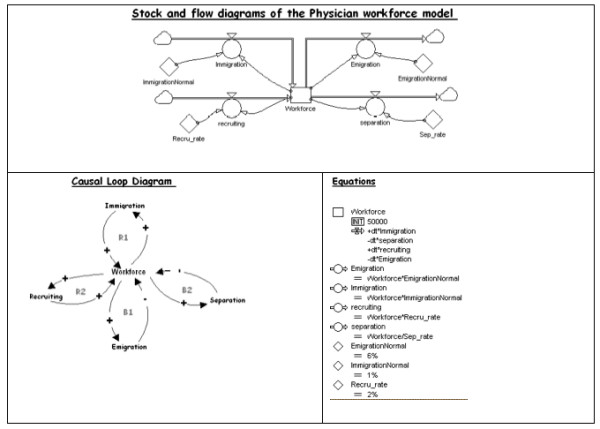
**Illustration of the elements of system dynamics model**. A simple model of physician workforce.

In order to illustrate the method, Figure 1 shows a medical workforce simple example of system dynamics with its basic elements: causal loops diagram, stock and flow diagrams and equations. Causal loops include feedback loops, reinforcing (+) and balancing (-). In the stock and flow diagram, system dynamics standard notation is used: stock variables are represented as squares, flow variables are circles and constants are diamonds. Equations represent mathematical relationships between variables.

The System Dynamics simulation model of medical specialists in Spain from 2008 to 2025 starts with the design of the theoretical model and its causal relations, the causal loop, to represent the most relevant aspects and determinants of the system as it operates. Once the variables, dependent and independent, have been identified and the relationship between them specified, the formal model, in the form of stock and flow diagrams, is drawn up using conventional System Dynamics notation--squares as stocks, pipe-like arrows as flows, circles as auxiliary variables, rhomboids as constants, and links as influences.

The structure of our model has two components: the submodel of supply and the submodel of demand/need. The base year is 2008 and the simulation is projected up to 2025 (See Additional file 1 for equations and Additional file 2 for input data).

### The submodel of supply

**Figure 2:**
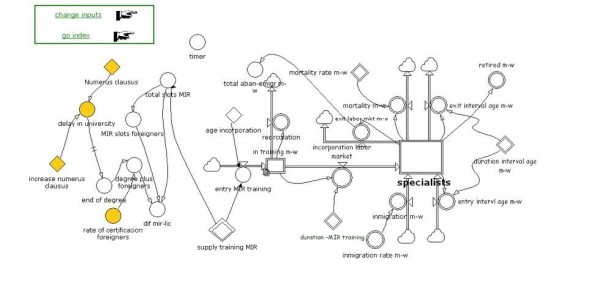
**Stock and flow diagrams. **Submodel of the supply of medical specialists 2008-2025. The number of doctors of each sex in each one of the 47 specialties depends on the new arrivals to the market (inmigration, training) and on the exits (retirements, drop-outs, mortality). In each step of the simulation the model shifts the medical population one year ahead, with 36 age-sex intervals (30 to 65 years of age). Age-sex pyramids for each specialty and year in the time horizon 2008-2025 are calculated.

The submodel of supply (Figure 2) shows the worklife cycle of physicians from training until retirement or death. The cycle begins with admission to university as medical students (in Spain there is no liberal arts or pre-med phase), for whom enrolment is limited to a maximum number, or numerus clausus, which is a parameter in the model. After six years of university classes, students have a degree (licenciatura) in general medicine. To be accepted into a training program to be a specialist, they must then pass a national examination which allows them to apply for one of the approximately 7000 training positions (another parameter) in 47 specialties, of which we considered 43, including family medicine, in accredited medical centres. This period, known as MIR training (intern resident physician), lasts four or five years, depending on the specialty.

The supply submodel was implemented for each of the 43 specialties, and separately for women and men, since the flows that affect the stock of specialists, emigration and immigration, drop-outs, productivity, mortality, etc., differ significantly by gender. Hence we applied the model vectorally for 43 × 2 submodels. We worked with 36 age groups (from 30 to 64 years of age), so that the model 'ages' annually the individuals in each age group and one can estimate the population pyramid of each specialty for any given year between 2008 and 2025.

In the supply submodel, the parameters the planner can manipulate each year in order to produce alternative scenarios are as follows: the number of students admitted to medical school; the number of residencies available for each specialty; the mandatory retirement age; the equivalent full-time ratio; and the immigration rate by specialty, which depends on the certification and regulation of foreign-trained physicians.

The baseline model assumes that all the controllable parameters will remain at their current values, except the number of admission places for medical students, which includes a planned increase.

### The submodel of demand/need

The demand/need submodel was based on normative standards of need for each specialty or group of specialties in the baseline year and over the successive years. The need for specialists in Spain in the baseline year was estimated from information on deficit (the positions unfilled) reported by authorities in the Autonomous Communities and those listed on the job market. Starting with this baseline year, the evolution of estimated future needs was based on a hypothetical growth rate of the appropriate ratio of specialists to 1000 population, with specialties divided into four groups according to level of demand (sharply increasing, moderately increasing, stable, decreasing) as judged by the panel of experts. The growth rates we used in the model are reported in Table 1, and are those used by the US Department of Health and Human Services [51].

**Table 1:** Growth rates for the demand/need for medical specialists, Spain, 2008-2025

	Annual per-capita growth rate	Cumulative 2008-2025 growth rate
Specialties w/sharply increasing demand	1.30%	24.50%

Specialties w/stable/increasing demand	0.60%	10.70%

Specialties w/stable demand	0.00%	0.00%

Specialties w/decreasing demand	-0.60%	-9.70%

These rates and appropriate standards can be set as parameters, as the model is an instrument that allows the Health Ministry to change them according to the evolution of the real system; for the great value of the model is its capacity to respond to hypothetical "What if...?" questions. On the demand side, the model allows the analysis of the degree of sensitivity of the parameters that are most uncertain: population growth (with scenarios for rapid, moderate, and slow), and the growth rate for the demand of each specialty. In the baseline model, a moderate growth rate has been assumed.

The main outputs of the model are, for each specialty and year, the number of specialists, their full-time equivalents, the demographic pyramid, the ratio for 100 000 inhabitants, the percentage of women, and the percentage of those under 51 years of age.

## Results

In the scenario of the baseline model with moderate population growth, the deficit of medical specialists will grow from 2% at present (2800 specialists) to 14.3% in 2025 (almost 21 000) (Table 2). With rapid population growth like that of the past five years, the tendency towards deficit would be much sharper, and the deficit of specialists would be twice a big as in the scenario with moderate growth, with a drop in the ratio of specialists per 100 000 population from 319 in 2008 to 305 in 2025. But even in a slow growth hypothesis there would be a deficit of 15 200 specialists, or 10.0%, in 2025.

**Table 2:** Baseline model. Scenario with moderate population growth

	2008	2015	2025
Inhabitants	44 366 332	46 333 661	48 018 184

Total medical specialists needed	141 579	149 563	152 160

Ratio specialists/100 000 inhab.	144 410	157 490	173 918

Deficit/surplus specialists (%)	-2.0%	-5.3%	-14.3%

**Figure 3:**
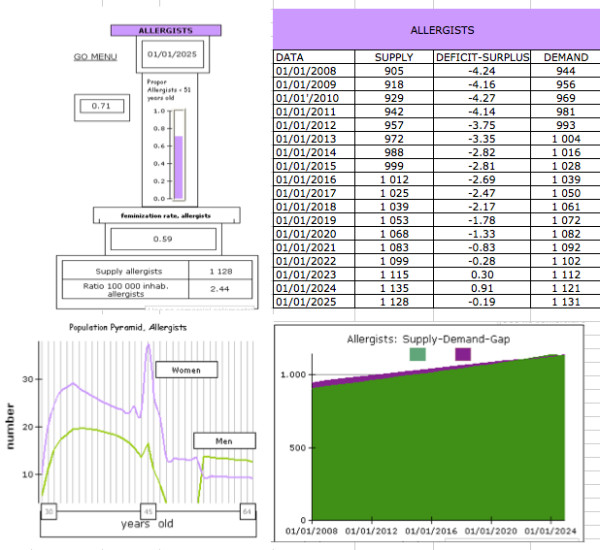
**Summarized model output up to 2025 for one specialty (allergists)**.

By specialty there would be significant differences in the trends of physician supply. The projections are largely based on the present number of specialists, the shape of estimated population pyramids (age and sex), and the number of residencies offered. The specialties with the oldest population pyramids, generally the most traditional and which have the lowest proportion of women, have the highest rates of decline in their supply, largely because of the greater rate of exit of specialists from the labour market. This effect is mitigated in those specialties in which there has been growth in the residencies offered and those which have younger population pyramids, which often correspond to those that have a high proportion of women (which in turn has an opposite effect because of their higher dropout and retirement rate). As an example, Figure 3 shows the output for allergists.

Under baseline parameters, the specialties with the greatest medium-term deficits are Anesthesiology (which in Spain does not include critical care), Orthopedic and Traumatic Surgery, Pediatric Surgery, Plastic Aesthetic and Reparatory Surgery, Family and Community Medicine, Pediatrics, Radiology, and Urology.

There will also be deficits, but less severe, in Vascular Medicine and Surgery, Gastroenterology, Cardiology, General Surgery, Thoracic Surgery, Endocrinology and Nutrition, Geriatrics, Neurosurgery, Obstetrics and Gynecology, Ophthalmology, Medical Oncology, Eye Ear Nose and Throat, Psychiatry and Rheumatology.

## Discussion

The methods and applications of System Dynamics and system feedback modeling for policy analysis can assist in designing better policies for the supply of physicians that take into account the complexity of social and ecological environments and a plurality of perspectives.

The main objective of our model was to simulate the consequences of different policies aimed at improving the capacity of the Spanish health system. Schools of Medicine take six years to 'produce' a physician, and the MIR system takes four to five additional years to train a specialist. From the point of view of the model, these are time delays that affect the behavior of the entire system. From the point of view of the planner, he has to make choices one decade before the effects of his policies start to be effective. Ideally, the model could treat the policy variables-numerus clausus, number of MIR positions-as functions of the estimated number of required health professionals, which in turn depends on the lagged choices, in a feedback loop. We decided instead to introduce those policy decisions as model parameters, because our model was design to be used by the planner to simulate the effect of potential changes in their choices. The model does not provide 'a solution', it is rather a tool to know "What would happen if...".

Although the model is a useful planning tool, as a way to simulate the effects of regulatory changes on the health sector it has its limitations. The supply submodel will be realistic in its conclusions to the extent that the entry parameters that govern its assumptions are realistic. Fortunately, the model and the software by which it is implemented allows the modification of these parameters--places for students in medical schools, number of residencies, mandatory retirement age, immigration, etc.-allowing the planner to see what would happen if the parameters under planning control were changed, whether one at a time or in combination. The planner would use the parameters as tools in human resource policy and to regulate the supply.

Another, greater, limitation is the lack of normative standards for the need of specialists, whether by population ratios or other measures. The way the deficit is calculated, based on empirical criterion of demand (number of unfilled positions), assumes implicitly that the present number of staff positions is appropriate.

The model assumes a given level of net immigration (entries minus exits) by specialty and year. Although immigration rates can be used as parameters, they are quite unpredictable, as they depend on international markets and underlying forces of push and pull [52]. State authorities, by the regulation of entry visas and certification, can only partially affect these parameters. Another limitation is that this is an isolated model, only for physicians, and it excludes other health professionals, such as nurses. An integral planning model for health professionals, as recommended by international organizations, would be preferable [53].

## Conclusions

In Spain there are deficits of doctors in certain specialties and zones, which will get worse in years to come for easily predictable reasons. These deficits can be due to two causes, those related to price control (the salaries and income of the professionals) and quantity control (barriers to entry into the profession and international mobility). In Spain the deficit of physicians, which varies substantially among specialties, is due to both causes.

We have identified current deficits in some specialties, which could worsen over the medium and long term or be mitigated by human resource policies that the model helps to pre-screen. It will not be easy, however, given the short-term lack of flexibility and capacity for adaptation of the supply of physicians, whose de facto mobility, whether within the country between Autonomous Communities or within the profession between specialties, is extremely limited. There is a persistent problem in the public health system's lack of capacity to attract good physicians for less attractive positions.

The model suggests the need to increase the number of students admitted to medical school, as Spain's neighbours have done in recent years. In the meantime, the need to make more flexible the supply in the short term is being filled by the immigration of physicians from new members of the European Union and from Latin America. Cultural diversity, which might enrich the health system and improve its efficacy with a more suitable assignment, say, of immigrant patients to doctors from their home countries, is not being taken advantage of.

The model already started to prove its usefulness in the planning practice in Spain. Its first version, issued in 2007, contributed to design some changes, particularly of the *numerus clausus *to medical schools and the number of training positions of medical specialists, by prioritizing those specialties with larger shortages. At present there is a Project for a Royal Decree on the homologation of the medical specialist degree from non EU-countries that tries to solve some of the problems indicated by our analysis.

## Competing interests

The authors declare that they have no competing interests.

## Authors' contributions

Both authors have contributed substantially to the design, data collection, analysis and discussion of results and have seen and approved its final version.

## Supplementary Material

Additional file 1 **Equations for the simulation model, "The need for medical specialists 2008-2025"**. Click here for file

Additional file 2 **Data file**. Click here for file
